# Prevalence of mental health symptoms and associated risk factors among healthcare workers in specialized COVID-19 hospitals in Anyang, China: A cross-sectional survey

**DOI:** 10.1016/j.heliyon.2024.e32593

**Published:** 2024-06-06

**Authors:** Ya-Hui Xu, Fang Wu, Shuai Yu, Xiao-Yang Zhang, Peng-Jiao Xu, Qi-Meng Sun

**Affiliations:** Department of Sleep Medicine, Second Affiliated Hospital of Xinxiang Medical University; Henan Collaborative Innovation Center for Prevention and Treatment of Mental Disorders; Brain Institute, Henan Academy of Innovations in Medical Science, Xinxiang, China

**Keywords:** COVID-19, Healthcare workers, Mental health, Prevalence, Risk factors

## Abstract

**Background:**

The novel coronavirus disease 2019 (COVID-19) pandemic spread worldwide and brought unprecedented challenges to healthcare systems. Healthcare workers experienced tremendous pressure and psychological issues.

**Methods:**

A cross-sectional online survey was conducted from January 2022 to April 2022 among healthcare workers in Anyang, Henan Province, China. Insomnia, anxiety, depression, post-traumatic stress disorder (PTSD), and problematic internet use (PIU) were evaluated. Logistic regression analyses were used to explore the factors that were associated with mental health problems.

**Results:**

A total of 242 participants (mean [SD] age, 34.7 [6.6] years, 187 female [77.3 %]) were included in the study. The prevalence of symptoms of insomnia, anxiety, depression, PTSD and PIU during the COVID-19 pandemic in China was 53.7 %, 100.0 %, 7.0 %, 20.3 %, and 19.4 %, respectively. Participants who smoked, used sedative-hypnotic drugs and may need psychological assistance were at a higher risk for mental health problems. Respondents who were older than 45 years and were married displayed a lower risk of insomnia and PTSD, respectively.

**Conclusions:**

Mental health symptoms are pervasive among healthcare workers in specialized COVID-19 hospitals during the outbreak. Risk factors include smoking, sedative-hypnotic drug use, and the need for psychological assistance, while protective factors include age and marital status. Developing social media platforms and providing psychological assistance may be effective interventions for healthcare workers.

## Highlights

• High prevalence of psychological distress was observed among healthcare workers during the COVID-19 pandemic.
• Identified risk factors include age, smoking, sedative-hypnotic drug use, and perceived need for psychological assistance.
• Targeted interventions, like smoking cessation and mental health resources, are essential for supporting healthcare workers.

## 1 Introduction

An outbreak of a novel transmissible respiratory disease COVID-19 was first reported in Wuhan, Hubei Province, China in December 2019 [[1], [2], [3], [4]]. A series of new SARS-CoV-2 genetic variants emerged and were recognized by World Health Organization (WHO) in January 2020 [5,6]. The SARS-CoV-2 Omicron (B.1.1.529) variant was first reported in South Africa on November 24, 2021, and fueled widespread fear due to its contagiousness and immune evasion capabilities [7]. Omicron is the fifth variant of concern (VOC) after Alpha, Beta, Gamma, and Delta [8]. As of October 25, 2023, there have been 771,549,718 confirmed cases of COVID-19, including 6,974,473 deaths reported to the WHO (https://covid19.who.int/). The worldwide COVID-19 pandemic has brought unprecedented challenges to healthcare systems.

A systematic review reported a rate of 51.7 % infection among healthcare workers during the first 6 months of the COVID-19 pandemic [9]. Healthcare workers are at higher risk of suffering from different kinds of adverse psychological outcomes while facing tremendous pressure from COVID-19 [10]. The Patient Centered Outcomes Research Institute registry cross-sectional survey reported that 53 % of US healthcare workers had emotional distress experiences [11]. A prospective cohort study exhibited that one-fifth of healthcare workers experienced psychological distress for 7 months following the COVID-19 pandemic surge [12]. A lack of rest time, mourning the death of friends or loved ones due to COVID-19 and personal COVID-19 status, healthcare workers had a higher incidence of psychosocial problems and risk for developing them [[13], [14], [15]]. During the COVID-19 lockdown, a progressive increase in problematic internet use (PIU) was observed [16], and it was significantly correlated to the unpleasant emotions [17]. Mental health problems led to burnout and discontinuity of healthcare workloads [18,19]. Therefore, psychological disorders among healthcare workers should receive increased attention during the COVID-19 outbreak.

The COVID-19 pandemic has markedly impacted the mental health of healthcare workers, with high prevalence rates of insomnia, stress, anxiety, and depression observed [20]. Amid the SARS-CoV-2 outbreak, healthcare workers encounter exacerbated psychological strain, potentially leading to mental illness [21]. The COVID-19 pandemic presents significant hazards and risks to the mental health of healthcare workers with mental disorders, including increased anxiety, depression, and psychological distress due to factors such as fear of infection, isolation measures, disrupted treatment, and social stigma [22]. Following the detection of 84 COVID-19 cases in Anyang, Henan, 5 million residents were placed under lockdown, making it the third city, after Xi'an and Yuzhou, to enforce such stringent measures. However, there has been limited research investigating the psychological challenges encountered by healthcare workers during the outbreak of the highly transmissible Omicron variant in China. Moving forward, it will be crucial to offer tailored psychological support to safeguard the well-being of healthcare workers, given the recent pandemic.

This cross-sectional online survey aims to assess the prevalence of psychological issues among healthcare workers during the COVID-19 pandemic, including insomnia, anxiety, depression, post-traumatic stress disorder (PTSD), and problematic internet use, while identifying associated risk factors to inform targeted interventions. The findings are crucial for developing tailored strategies to support healthcare workers' psychological well-being, ensure effective healthcare delivery amid the ongoing pandemic, and provide evidence-based guidance for future crisis responses.

## 2 Methods

### 2.1 Study design

This is a cross-sectional online survey with convenience sampling conducted from January 2022 to April 2022 in Anyang, Henan Province, China. A self-report questionnaire was designed to investigate the mental health status of healthcare workers during a period when the cumulative cases of COVID-19 reached a peak in Anyang. The data were anonymously collected through self-response questionnaires using a platform called Questionnaire Star (www.wjx.cn).

### 2.2 Participants

The data were collected from the Fifth People's Hospital of Anyang (a specialized COVID-19 hospital). A total of 242 participants took part in the study and successfully submitted the questionnaire. The inclusion criteria were as follows: (a) aged 18–65 years; (b) healthcare workers; (c)willing to participate in the survey; and (d) no apparent cognitive impairment. The exclusion criteria were: (a) not meeting the inclusion criteria; (b) incomplete data on the online questionnaire; and (c) unable to participate due to psychological problems. Oral informed consent was obtained at the beginning of the questionnaire, and participants were considered to have provided consent by default if they completed the survey.

### 2.3 Measurements

The survey lasted approximately 10 min and gathered information in four domains:

(1) demographic characteristics, including age, gender, level of education, marital status, occupation, hazardous drinking and smoking; (2) insomnia symptoms were assessed by the Pittsburgh Sleep Quality Index (PSQI), and sedative-hypnotic drugs usage-related information was assessed by asking the following questions: Have you ever used sedative-hypnotic drugs? Whether you will ask the doctor about the usage and dosage of insomnia drugs and precautions? Have you increased the dosage of medication to increase the efficacy? Whether you stopped or reduced your medication because your symptoms improved or had no effect? The response options for each of the four questions above were “yes” or “no”. (3) psychological symptoms including anxiety, depression, post-traumatic stress disorder and problematic internet use were evaluated by the Beck Anxiety Inventory (BAI), the Beck Depression Inventory-IA (BDI-IA), the PTSD Short Screening Scale (PTSD-7) and the Young Internet Addiction Scale. (4) psychological assistance information by asking the following questions: Do you think psychological assistance is necessary? The response options were “yes” or “no”. The main mental health outcomes were insomnia, anxiety, depression, post-traumatic stress disorder and problematic internet use.

### 2.4 Insomnia

The Pittsburgh Sleep Quality Index (PSQI) was used to evaluate sleep quality and sleep disturbances over one month [23,24]. The PSQI questionnaire consists of 19 questions that represent seven components: (1) subjective sleep quality, (2) sleep latency, (3) sleep duration, (4) sleep efficiency, (5) sleep disturbance, (6) daytime dysfunction and (7) sleep medication intake. Each component is scored from 0 to 3. The total score is extended from 0 to 21, with lower scores indicating greater sleep quality. Symptom severity was interpreted as follows: minimal (≤5), mild (5–10), moderate (11–15), and severe (16–21) insomnia. PSQI scores above a cut-off of 5 are indicated clinically significant insomnia.

### 2.5 Anxiety

The Beck Anxiety Inventory (BAI) is a structured self-reported questionnaire used to evaluate the intensity of anxiety symptoms in the previous week [25,26]. It consists of 21 items that investigate common symptoms of anxiety, such as numbness or tingling, dizziness, lightheadedness, nervousness, terror, fear of dying, and sweating. The responses were rated on a 4-point Likert scale ranging from 0 (none) to 3 (severe). The total scores range from 0 to 63, with higher scores indicating higher anxiety levels experienced by the individual. Overall total scores are classified into four categories, including minimal (≤9), mild (10–18), moderate (19–29), and severe (30–63) anxiety levels. A total score above 29 indicated severe anxiety.

### 2.6 Depression

The Beck Depression Inventory-IA (BDI-IA) is a 21-item self-report inventory used to measure the presence and severity of depression in the past two weeks [27,28]. This scale assessed somatic, affective, cognitive, and vegetative symptoms. Each statement is assigned a score ranging from 0 to 3, and the total scores range from 0 to 63. The standard severity ranges are as follows: minimal (≤9), mild (10–18), moderate (19–29), and severe (30–63) depression levels. A total score above 18 indicated significant symptoms of depression.

### 2.7 Post-traumatic stress disorder

Post-traumatic stress disorder (PTSD) was assessed using the PTSD Short Screening Scale (PTSD-7) for Diagnostic and Statistical Manual of Mental Disorders (Fourth Edition) PTSD [29,30]. The PTSD-7 consists of seven items assessing elevated arousal and symptoms of avoidance. A score of four or more on the short screening scale was used to identify positive cases of PTSD [31].

### 2.8 Problematic internet use

The Young Internet Addiction Scale was used for the assessment of problematic internet use (PIU), which includes 20 items with a total score from 20 to 100 [32]. The total scores below 40 indicate average online users, 40–69 signifies problematic users, 70 and above reflect significant problematic users [33]. A score of 40 or above is considered as problematic internet use.

### 2.9 Statistical analysis

Descriptive statistics analysis was used to characterize the total sample and pandemic-related information. The prevalence of symptoms of insomnia, anxiety, depression, acute stress and problematic internet use was reported as the percentages of cases in all populations. PSQI, BDI-IA, BAI, PTSD-7 and Young Internet Addiction Scale scores were treated as dichotomous variables. Univariate logistic regression analyses were initially used to assess the association between outcome variables and potential factors. A univariate association with p-value less than to 0.1 was included in the multivariate logistic regression analysis. Multicollinearity was assessed through variance inflation factors (VIFs). Explanatory variables with VIF values surpassing the threshold of 10 were identified as exhibiting multicollinearity and consequently excluded from the model. Odds ratios (ORs) and 95 % confidence intervals (CIs) were determined for the logistic regression analyses. The level of significance was set to P < 0.05 (two-tailed). All of the statistical analyses were conducted using IBM SPSS Statistics 26.0.

## 3 Results

### 3.1 Demographic characteristics

In this cross-sectional online survey, 242 participants ranging from 18 to 65 years old with a mean (SD) age of 34.7 (6.6) were recruited in the present study. Of the participants, the majority were female 187 (77.3 %). 175 (72.3 %) were married, 210 (86.8 %) had a university degree or higher. Only a small portion of participants were drinking (29 [12.0 %]), smoking (16 [6.6 %]), 44 (18.2 %) having used sedative-hypnotic drugs, 12 (5.0 %) having increased the dose of medications to increase the efficacy, and 23 (9.5 %) having stopped or reduced the medication because their symptoms improved or had no effect. Of the total number of respondents, 127 (52.5 %) may ask the doctor about the usage and precautions of insomnia medications, 108 (44.6 %) may need psychological assistance. Other details of the demographic characteristics are shown in Table 1.

**Table 1:** Demographic characteristics of the total sample.

Factor	Modality	Participants
**Overall**		242 (100.0 %)
**Age (years)**		
	18–30 years	54 (22.3 %)
	31–45 years	163 (67.4 %)
	46–65 years	15 (6.2 %)
	Missing	10 (4.1 %)
**Gender**		
	Male	55 (22.7 %)
	Female	187 (77.3 %)
**Education level**		
	Technical secondary or below	32 (13.2 %)
	Bachelors	153 (63.2 %)
	Masters or above	57 (23.6 %)
**Marital status**		
	Unmarried	54 (22.3 %)
	Married	175 (72.3 %)
	Separated/divorced	13 (5.4 %)
**Drinking**		
	No	213 (88.0 %)
	Yes	29 (12.0 %)
**Smoking**		
	No	226 (93.4 %)
	Yes	16 (6.6 %)
**Have you used sedative-hypnotic drugs?**		
	No	198 (81.8 %)
	Yes	44 (18.2 %)
**Whether you will ask the doctor about the usage and precautions of insomnia medications?**		
	No	115 (47.5 %)
	Yes	127 (52.5 %)
**Have you increased the dosage of medications to increase the efficacy?**		
	No	230 (95.0 %)
	Yes	12 (5.0 %)
**Have you stopped or reduced the dose of medications because your symptoms improved or had no effec**t？		
	No	219 (90.5 %)
	Yes	23 (9.5 %)
**Do you think psychological assistance is necessary?**		
	No	134 (55.4 %)
	Yes	108 (44.6 %)

### 3.2 Prevalence of symptoms of insomnia, depression, anxiety, PTSD, problematic internet use

Table 2 presents the prevalence of the overall psychological problems in the total sample. For insomnia, a total of 130 (53.7 %) respondents reported PSQI scores ≥5, including 99 (40.9 %) with mild insomnia, and 31 (12.8 %) with moderate to severe insomnia. The scores on the BAI showed that the largest proportion of participants experienced moderate to severe anxiety (83.5 % and 16.5 %, respectively). The total prevalence for depression symptoms was 7.0 %, while 5.8 % reported having moderate depression symptoms and 1.2 % severe depression symptoms. The prevalence rates of PTSD symptoms and problematic internet use was 20.3 % and 19.4 %, respectively. The highest prevalence of psychological problems was anxiety (100.0 %) and the lowest prevalence was depression (7.0 %).

**Table 2:** Prevalence of symptoms of Insomnia, Depression, Anxiety, PTSD, problematic internet use.

Factor	Modality	Participants
Insomnia		
	normal (0–5)	112 (46.3 %)
	mild (6–10)	99 (40.9 %)
	moderate (11–15)	27 (11.2 %)
	severe (16–21)	4 (1.7 %)
**Anxiety**		
	normal (0–9)	0 (0.0 %)
	mild (10–18)	0 (0.0 %)
	moderate (19–29)	202 (83.5 %)
	severe (30–63)	40 (16.5 %)
**Depression**		
	normal (0–9)	183 (75.6 %)
	mild (10–18)	42 (17.4 %)
	moderate (19–29)	14 (5.8 %)
	severe (30–63)	3 (1.2 %)
**Post-Traumatic Stress Disorder (PTSD)**		
	No (0–3)	193 (79.8 %)
	Yes (4–7)	49 (20.3 %)
**Problematic internet use(PIU)**		
	normal (0–39)	195 (80.6 %)
	problematic users (40–69)	43 (17.8 %)
	severe (70–100)	4 (1.7 %)

### 3.3 Factors associated with mental health symptoms

The correlations between potential risk factors for psychological problems were analyzed by binary logistic regression (Table 3). The results of the unadjusted analysis of demographic and relevant variables showed that several factors were independently associated with insomnia (PSQI score ≥5), severe anxiety (BAI score ≥30), depression (BDI score ≥19), post-traumatic stress (PTSD score ≥4) and problematic internet use (Young Internet Addiction Scale score ≥40).

**Table 3:** Univariable regression analysis of factors associated with psychological problems.

		Insomnia	*P*	Anxiety	*P*	Depression	*P*	PTSD	*P*	PIU	*P*
**Age**		OR (95 % CI)		OR (95 % CI)		OR (95 % CI)		OR (95 % CI)		OR (95 % CI)	
	18–30 years	1 [Reference]		1 [Reference]		1 [Reference]		1 [Reference]		1 [Reference]	
	31–45 years	0.519(0.273–0.988)	**0.046**	0.776(0.356–1.694)	0.524	0.849(0.288–2.503)	0.767	0.611(0.299–1.245)	0.175	0.741(0.355–1.547)	0.424
	46–65 years	0.182(0.051–0.652)	**0.009**	0.279(0.033–2.359)	0.241	0.700(0.075–6.495)	0.754	0.400(0.080–1.988)	0.263	0.788(0.192–3.231)	0.741
**Gender**											
	Male	1 [Reference]		1 [Reference]		1 [Reference]		1 [Reference]		1 [Reference]	
	Female	1.398(0.765–2.554)	0.276	1.213(0.523–2.811)	0.653	1.622(0.455–5.784)	0.456	0.914(0.439–1.901)	0.810	1.304(0.587–2.896)	0.515
**Education level**											
	Technical secondary or below	1 [Reference]		1 [Reference]		1 [Reference]	0.310	1 [Reference]	0.207	1 [Reference]	0.268
	Bachelors	1.445(0.671–3.113)	0.347	0.672(0.273–1.651)	0.386	0.650(0.197–2.141)	0.479	0.890(0.367–2.155)	0.796	0.793(0.326–1.932)	0.610
	Masters or above	0.407(0.167–0.993)	**0.048**	0.226(0.062–0.825)	**0.024**	0.255(0.044–1.476)	0.127	0.420(0.136–1.294)	0.131	0.420(0.136–1.294)	0.131
**Marital status**											
	Unmarried	1 [Reference]		1 [Reference]		1 [Reference]		1 [Reference]		1 [Reference]	
	Married	0.537(0.285–1.010)	0.054	1.095(0.467–2.569)	0.611	0.721(0.242–2.148)	0.558	0.491(0.243–0.994)	**0.048**	0.875(0.407–1.880)	0.732
	Separated/divorced	0.869(0.249–3.029)	0.825	2.556(0.632–10.330)	0.209	1.782(0.305–10.413)	0.521	1.056(0.283–3.930)	0.936	1.737(0.450–6.710)	0.423
**Drinking**											
	No	1 [Reference]		1 [Reference]		1 [Reference]		1 [Reference]		1 [Reference]	
	Yes	2.081(0.906–4.778)	0.084	0.787(0.258–2.398)	0.673	0.387(0.050–3.012)	0.364	1.258(0.504–3.138)	0.623	1.700(0.701–4.119)	0.240
**Smoking**											
	No	1 [Reference]		1 [Reference]		1 [Reference]		1 [Reference]		1 [Reference]	
	Yes	4.037(1.120–14.552)	**0.033**	2.481(0.812–7.579)	0.111	0.770(0.096–6.171)	0.806	3.310(1.168–9.384)	**0.024**	1.991(0.657–6.036)	0.223
**Have you used sedative-hypnotic drugs?**											
	No	1 [Reference]		1 [Reference]		1 [Reference]		1 [Reference]		1 [Reference]	
	Yes	3.612(1.693–7.710)	**0.001**	2.656(1.237–5.704)	**0.012**	1.220(0.384–3.871)	0.736	1.825(0.871–3.823)	0.111	2.023(0.960–4.263)	0.064
**Whether you will ask the doctor about the usage and precautions of insomnia medications?**											
	No	1 [Reference]		1 [Reference]		1 [Reference]		1 [Reference]		1 [Reference]	
	Yes	0.985(0.594–1.634)	0.954	0.547(0.274–1.090)	0.086	0.389(0.143–1.060)	0.065	0.651(0.348–1.218)	0.179	0.547(0.286–1.044)	0.067
**Have you increased the dosage of medications to increase the efficacy?**											
	No	1 [Reference]		1 [Reference]		1 [Reference]		1 [Reference]		1 [Reference]	
	Yes	10.261(1.303–80.777)	**0.027**	3.980(1.195–13.249)	**0.024**	2.506(0.508–12.369)	0.259	2.937(0.891–9.681)	0.077	6.650(2.009–22.017)	**0.002**
**Have you stopped or reduced the dose of medications because your symptoms improved or had no effe**ct？											
	No	1 [Reference]		1 [Reference]		1 [Reference]		1 [Reference]		1 [Reference]	
	Yes	6.606(1.908–22.875)	**0.003**	2.466(0.942–6.455)	0.066	1.903(0.510–7.095)	0.338	4.231(1.740–10.286)	**0.001**	2.462(0.976–6.209)	0.056
**Do you think psychological assistance is necessar**y？											
	No	1 [Reference]		1 [Reference]		1 [Reference]		1 [Reference]		1 [Reference]	
	Yes	1.719(1.028–2.875)	**0.039**	1.649(0.833–3.262)	0.151	2.919(1.071–7.960)	**0.036**	2.192(1.162–4.136)	**0.015**	1.533(0.809–2.906)	0.190

Note: PSQI score ≥5 indicates insomnia; BAI score ≥30 indicates severe anxiety; BDI score ≥19 indicates depression; PTSD score ≥4 indicates post-traumatic stress; Young Internet Addiction Scale score ≥40 indicates problematic internet use.

Abbreviation: PIU: problematic internet use.

In the multivariate logistic regression analysis (Table 4), being older than 45 years (OR = 0.155, 95 % CI 0.038 to 0.639, p = 0.010), smoking (OR = 4.786, 95 % CI 1.224 to 18.716, p = 0.024), having used sedative-hypnotic drugs (OR = 2.785, 95 % CI 1.153 to 6.726, p = 0.023), having stopped or reduced the dose of medications (OR = 5.671, 95 % CI 1.414 to 22.74, p = 0.014) were significantly correlated with insomnia. Respondents having used sedative-hypnotic drugs (OR = 2.744, 95 % CI 1.266 to 5.947, p = 0.011) were associated with severe anxiety. Nonetheless, individuals who may ask the doctor about the usage and precautions of insomnia medications (OR = 0.358, 95 % CI 0.130 to 0.989, p = 0.048) had a lower risk for depression. Smoking (OR = 3.704, 95 % CI 1.246 to 11.013, p = 0.019), having stopped or reduced the dose of medications (OR = 3.926, 95 % CI 1.558 to 9.892, p = 0.004) were susceptible to symptoms of post-traumatic stress disorder. Participants who were married (OR = 0.458, 95 % CI 0.218 to 0.962, p = 0.039) had a lower risk of PTSD. Having increased the dose of medications to increase the efficacy (OR = 9.085, 95 % CI 2.614 to 31.572, p = 0.001) was statistically significantly associated with a higher risk of problematic internet use. However, those who may ask the doctor about the usage and precautions of insomnia medications (OR = 0.456, 95 % CI 0.229 to 0.908, p = 0.025) were independently associated with a lower risk of problematic internet use. Participants who may need psychological assistance had a higher risk of depression (OR = 3.144, 95 % CI 1.140 to 8.672, p = 0.027) and PTSD (OR = 1.963, 95 % CI 1.001 to 3.852, p = 0.050).

**Table 4:** Multivariable regression analysis of factors associated with psychological problems.

		Insomnia	*P*	Anxiety	*P*	Depression	*P*	PTSD	*P*	PIU	*P*
**Age(reference: 18**–**30 years)**		**OR (95 % CI)**		**OR (95 % CI)**		**OR (95 % CI)**		**OR (95 % CI)**		**OR (95 % CI)**	
	31–45 years	0.766(0.372–1.577)	0.470								
	46–65 years	0.155(0.038–0.639)	**0.010**								
**Education level(reference: Technical secondary or below)**											
	Bachelors	1.739(0.744–4.065)	0.202								
	Masters or above	0.432(0.154–1.216)	0.112								
**Marital status (reference: Unmarried)**											
	Married							0.458(0.218–0.962)	**0.039**		
	Separated/divorced							1.190(0.296–4.779)	0.806		
**Smoking (reference: no)**											
	Yes	4.786(1.224–18.716)	**0.024**					3.704(1.246–11.013)	**0.019**		
**Have you used sedative-hypnotic drugs? (reference: no)**											
	Yes	2.785(1.153–6.726)	**0.023**	2.744(1.266–5.947)	**0.011**						
**Whether you will ask the doctor about the usage and precautions of insomnia medications? (reference: no)**											
	Yes			0.527(0.261–1.063)	0.074	0.358(0.130–0.989)	**0.048**			0.456(0.229–0.908)	**0.025**
**Have you increased the dose of medications to increase the efficacy?(reference: no)**											
	Yes									9.085(2.614–31.572)	**0.001**
**Have you stopped or reduced the dose of medications because your symptoms improved or had no effect**？**(reference: no)**											
	Yes	5.671(1.414–22.740)	**0.014**					3.926(1.558–9.892)	**0.004**		
**Do you think psychological assistance is necessary? (reference: no)**											
	Yes					3.144(1.140–8.672)	**0.027**	1.963(1.001–3.852)	**0.050**		

Note: PSQI score ≥5 indicates insomnia; BAI score ≥30 indicates severe anxiety; BDI score ≥19 indicates depression; PTSD score ≥4 indicates post-traumatic stress; Young Internet Addiction Scale score ≥40 indicates problematic internet use.

Abbreviation: PIU: problematic internet use.

## 4 Discussion

The present cross-sectional survey investigated the prevalence of and factors associated with psychological symptoms during the COVID-19 pandemic. Most healthcare workers were married and held bachelor's degrees or higher. The prevalence of anxiety and insomnia (100.0 % and 53.7 %, respectively) was higher than that of post-traumatic stress (20.3 %), problematic internet use (19.4 %) and depression (7.0 %). Several factors associated with psychological problems were further identified, including age, education level, marital status, occupation, having used sedative-hypnotic drugs, may not ask the doctor about the usage and precautions of insomnia medications, having increased the dose of medications, having stopped or reduced the dose of medications, and those who may need psychological assistance. These findings provide targeted countermeasures for stratified psychological prevention and intervention strategies under pandemic conditions.

The present online survey revealed high rates of insomnia, anxiety, depression, acute stress and internet addiction in healthcare workers during the COVID-19 pandemic. The Emotional Epidemic Curve Model illustrates emotional and behavioral fluctuations during pandemics, featuring two peaks: one linked to misinformation and fear, and another to profound losses and social upheaval, impacting mental health significantly [34]. A large-scale meta-analytic atlas covering 32 countries provided a paramount picture of the high prevalence of mental disorders globally [35]. The prevalence of symptoms of insomnia, anxiety, depression, post-traumatic stress disorder, and psychological distress among populations was 23.87 %, 15.15 %, 15.97 %, 21.94 % and 13.29 %, respectively [36]. Healthcare workers have a significant prevalence in a wide range of mental health domains compared with non-medical health workers [[37], [38], [39], [40], [41]]. A previous study reported that the prevalence of anxiety, depression, and psychological distress was 38.12 %, 34.31 % and 37.54 %, respectively [42]. Our study was conducted during the Omicron outbreak period, which may have had a significant impact on the psychological well-being of healthcare workers. The findings are consistent with previous research indicating that being exposed to patients may increase the risk of severe emotional distress and there is an urgent need to develop coping strategies for healthcare workers during a pandemic.

In the present study, the prevalence of anxiety was 100.0 %, and insomnia was observed in 53.7 % of healthcare workers. The high prevalence of anxiety among healthcare workers may be attributed to the stressful work environment and the subjective nature of self-reported assessments. The prevalence of anxiety symptoms among undergraduate and graduate students was 24 % and 14 %, respectively [43]. This is consistent with our findings that anxiety is inversely associated with education level. The applicability of anxiety assessment tools varies across different groups and contexts. Specialized tools, tailored for children [44,45], women [46,47], healthcare workers [48,49], and during disasters or pandemics [50,51], offer more nuanced insights into anxiety levels and enable targeted interventions and support. Insomnia is ubiquitous comorbid with various psychiatric disorders [[52], [53], [54]]. Patients with insomnia and higher doses of sedative-hypnotic prescriptions exhibited higher risks of developing psychiatric disorders [55]. There is a bidirectional relationship between insomnia and anxiety [56], depression [57], PTSD [58]. A prominent increase in rates of sedative-hypnotic prescription was found during the COVID-19 pandemic [59,60]. Benzodiazepines play an important role in anxiety disorders [61], but patients who are on long-term treatment develop tolerance and need to increase the dosage of benzodiazepines to maintain efficacy. These observations can explain that those who increase the dose to improve the efficacy have a higher risk of anxiety. Similar to our findings, an online survey in India also found that younger individuals have a higher risk of insomnia than older individuals [62]. These findings may be related to occupational exhaustion in healthcare workers and underscore the importance of providing online psychological assistance for their catharsis during the COVID-19 pandemic outbreak.

It is noteworthy that a higher risk of post-traumatic stress, problematic internet use, and depression was also found in this study. A large proportion of healthcare workers were at risk of developing PTSD during the pandemic [[63], [64], [65]]. There is evidence that marriage represents a protective factor against adverse outcomes of post-traumatic stress [66]. A myriad of problematic use of internet has been linked to adverse mental health outcomes [67]. Young individuals were more likely to experience PIU compared to older individuals [68]. A population-based nationwide study in Bangladesh reported that the prevalence of depression and suicidal ideation was 33 % and 5 % [69]. Job burnout was positively associated with anxiety or depression among healthcare workers at the outbreak of COVID-19 [70]. These findings suggest that positive cognitive efforts and social support are required to lower rates of psychological distress.

Another intriguing finding in this study was that smoking and those who may need psychological assistance were associated with a higher risk of psychological problems. Findings indicated that smoking is significantly related to anxiety [71], insomnia [72] and post-traumatic stress [73]. Encouraging smoking cessation and reducing environmental smoke are important for mental health during COVID-19 times [74]. Protective factors such as age and marital status were also identified, suggesting potential associations with resilience or coping strategies [75]. A systematic literature review showed only 27 % of the studies included empirical evaluation of the digital interventions to assist healthcare workers [76]. An online psychological support package showed high usability and practicality among healthcare workers [77]. Developing social media platforms and providing psychological resources can help healthcare workers release stress.

Establishing tailored mental health support programs for healthcare workers is imperative. The proposed multi-level intervention framework, which targets individuals, organizations, and society, advocates for implementing resilience training, meeting basic needs, offering specialized job role training, enhancing leadership communication, addressing moral injury, fostering peer and social support, and normalizing mental health services [78]. Additionally, the Mental Health Preparedness and Action Framework suggests strategies such as preparation, monitoring, reducing misinformation-induced distress, sustaining mental health services, and effective communication [34]. Addressing mental health challenges for healthcare workers during COVID-19, the review and theoretical framework propose solutions including support recognition, clear guidance, improved testing, and addressing staffing shortages [79]. Measures should vary across different medical settings such as emergency departments, intensive care units, wards, mental health units, community health centers, academic institutions, and remote services. Dynamic interventions across 16 countries suggest alternating between strict COVID-19 measures and relaxation, anticipating reducing Intensive Care Unit admissions and deaths while alleviating critical care overload and global economic hardships [80]. Policymakers and managers need to prioritize the mental health of healthcare workers and implement supportive, encouraging, motivational, protective, and training and educational interventions [21].

### 4.1 Limitations

This study had several limitations. First, because of the temporality of the pandemic, this study adopted a cross-sectional design. Further research is needed to investigate the longitudinal trajectories of mental health outcomes among healthcare workers associated with the COVID-19 pandemic. Second, the inherent bias in online surveys using convenience sampling and reliance on self-report questionnaires rather than clinical diagnosis may limit the representativeness of the sample. Therefore, addressing biases like social desirability, self-perception, recall, selective participation, nonresponse, and common method biases is imperative for future research endeavors. Third, sample size is relatively small. Future larger-scale studies are needed to explore risk factors associated with mental health outcomes.

## 5 Conclusion

In conclusion, healthcare workers had a relatively high prevalence of insomnia, anxiety, depression, post-traumatic stress disorder and problematic internet use during the COVID-19 pandemic in the present study. Factors such as smoking, needing psychological assistance, having used sedative-hypnotic drugs, not consulting the doctor about the usage and precautions of insomnia medications, increasing the dose of medications, and stopping or reducing the dose of medications were associated with negative mental health outcomes. Protective factors such as age and marital status were also observed. The development of social media platforms and psychological assistance may reduce adverse psychological effects. Future research is needed to investigate long-term psychological outcomes and explore specific targeted interventions for healthcare workers.

## Ethical approval

The study was approved by the Ethics Board at Second Affiliated Hospital of Xinxiang Medical University (No. XYEFYLL-keyan-2022-01-3).

## Funding statement

This work was supported by the 10.13039/501100001809National Natural Science Foundation of China (no. 81601160), Open project of Henan Collaborative Innovation Center of Prevention and treatment of mental disorder, the Second Affiliated Hospital of Xinxiang Medical University (XTkf06) and grant from Henan Province Science and Technology Research Program Projects (no. 232102310109).

## Data availability statement

Data used in the present study can be accessed upon request from the corresponding authors.

## CRediT authorship contribution statement

**Ya-Hui Xu:** Writing – review & editing, Conceptualization. **Fang Wu:** Writing – original draft. **Shuai Yu:** Investigation, Data curation. **Xiao-Yang Zhang:** Methodology. **Peng-Jiao Xu:** Investigation. **Qi-Meng Sun:** Writing – original draft.

## Declaration of competing interest

The authors declare that they have no known competing financial interests or personal relationships that could have appeared to influence the work reported in this paper.
