# Supplementary material for: Prevalence of mental health symptoms and associated risk factors among healthcare workers in specialized COVID-19 hospitals in Anyang, China: A cross-sectional survey
Source: Heliyon. 2024 Jun 6;10(11):e32593. doi: 10.1016/j.heliyon.2024.e32593 (PMC11219988; doi:10.1016/j.heliyon.2024.e32593)
Supplement: Multimedia component 1 [file mmc1.doc]

Supplementary Questionnaire Information

**Assessment of the psychological states of frontline healthcare workers during the COVID-19 pandemic**

**Demographic characteristics**

1. What is your age?

2. Gender

□ Male

□ Female

3. Education level

□ Technical secondary or below

□ Bachelors

□ Masters or above

4. Marital status

□ Unmarried

□ Married

□ Separated/divorced

5. Drinking

□ Yes

□ No

6. Smoking

□ Yes

□ No

7. Have you used sedative-hypnotic drugs?

□ Yes

□ No

8. Whether you will ask the doctor about the usage and precautions of insomnia medications?

□ Yes

□ No

9. Have you increased the dosage of medications to increase the efficacy?

□ Yes

□ No

10. Have you stopped or reduced the dose of medications because your symptoms improved or had no effect？

□ Yes

□ No

11. Do you think psychological assistance is necessary?

□ Yes

□ No

**Pittsburgh Sleep Quality Index (PSQI)**

1. During the past month, what time have you usually gone to bed at night? ___________________

2. During the past month, how long (in minutes) has it usually taken you to fall asleep each night? __________

3. During the past month, what time have you usually gotten up in the morning? ___________________

4. During the past month, how many hours of actual sleep did you get at night? (This may be different than the number of hours you spent in bed.) ___________________

5. During the past month, how often have you had trouble sleeping because you...

a. Cannot get to sleep within 30 minutes

□ Not during the past month

□ Less than once a week

□ Once or twice a week

□ Three or more times a week

b. Wake up in the middle of the night or early morning

□ Not during the past month

□ Less than once a week

□ Once or twice a week

□ Three or more times a week

c. Have to get up to use the bathroom

□ Not during the past month

□ Less than once a week

□ Once or twice a week

□ Three or more times a week

d. Cannot breathe comfortably

□ Not during the past month

□ Less than once a week

□ Once or twice a week

□ Three or more times a week

e. Cough or snore loudly

□ Not during the past month

□ Less than once a week

□ Once or twice a week

□ Three or more times a week

f. Feel too cold

□ Not during the past month

□ Less than once a week

□ Once or twice a week

£Three or more times a week

g. Feel too hot

□ Not during the past month

□ Less than once a week

□ Once or twice a week

□ Three or more times a week

h. Have bad dreams

□ Not during the past month

□ Less than once a week

□ Once or twice a week

□ Three or more times a week

1. Have pain

□ Not during the past month

□ Less than once a week

□ Once or twice a week

□ Three or more times a week

j. Other reason(s), please describe: ___________________

How often during the past month have you had trouble sleeping because of this?

□ Not during the past month

□ Less than once a week

□ Once or twice a week

□ Three or more times a week

1. During the past month, how would you rate your sleep quality overall?

□ Very good

□ Fairly good

□ Fairly bad

□ Very bad

7. During the past month, how often have you taken medicine to help you sleep (prescribed or “over the counter”)?

□ Not during the past month

□ Less than once a week

□ Once or twice a week

□ Three or more times a week

8. During the past month, how often have you had trouble staying awake while driving, eating meals, or engaging in social activity?

□ Not during the past month

□ Less than once a week

□ Once or twice a week

□ Three or more times a week

9. During the past month, how much of a problem has it been for you to keep up enough enthusiasm to get things done?

□ No problem at all

□ Only a very slight problem

□ Somewhat of a problem

□ A very big problem

10. Do you have a bed partner or room mate?

□ No bed partner or room mate

□ Partner/room mate in other room

□ Partner in same room, but not same bed

□ Partner in same bed

If you have a room mate or bed partner, ask him/her how often in the past month you have had...

1. Loud snoring

□ Not during the past month

□ Less than once a week

□ Once or twice a week

□ Three or more times a week

1. Long pauses between breaths while asleep

□ Not during the past month

□ Less than once a week

□ Once or twice a week

□ Three or more times a week

1. Legs twitching or jerking while you sleep

□ Not during the past month

□ Less than once a week

□ Once or twice a week

□ Three or more times a week

1. Episodes of disorientation or confusion during sleep

□ Not during the past month

□ Less than once a week

□ Once or twice a week

□ Three or more times a week

e. Other restlessness while you sleep, please describe: ___________________

□ Not during the past month

□ Less than once a week

□ Once or twice a week

□ Three or more times a week

**Beck Anxiety Inventory (BAI)**

|  | Not At All | Mildly but it didn’t  bother me much | Moderately - it wasn’t  pleasant at times | Severely - it bothered me a lot |
| --- | --- | --- | --- | --- |
| Numbness or tingling | □ | □ | □ | □ |
| Feeling hot | □ | □ | □ | □ |
| Wobbliness in legs | □ | □ | □ | □ |
| Unable to relax | □ | □ | □ | □ |
| Fear of worst happening | □ | □ | □ | □ |
| Dizzy or lightheaded | □ | □ | □ | □ |
| Heart pounding/racing | □ | □ | □ | □ |
| Unsteady | □ | □ | □ | □ |
| Terrified or afraid | □ | □ | □ | □ |
| Nervous | □ | □ | □ | □ |
| Feeling of choking | □ | □ | □ | □ |
| Hands trembling | □ | □ | □ | □ |
| Shaky / unsteady | □ | □ | □ | □ |
| Fear of losing control | □ | □ | □ | □ |
| Difficulty in breathing | □ | □ | □ | □ |
| Fear of dying | □ | □ | □ | □ |
| Scared | □ | □ | □ | □ |
| Indigestion | □ | □ | □ | □ |
| Faint / lightheaded | □ | □ | □ | □ |
| Face flushed | □ | □ | □ | □ |
| Hot/cold sweats | □ | □ | □ | □ |

**Beck Depression Inventory****-IA (BDI-IA)**

1. Mood

□ I do not feel sad

□ I feel blue or sad

□ I am blue or sad all the time and I can't snap out of it

□ I am so sad or unhappy that I can't stand it

2. Pessimism

□ I am not particularly pessimistic or discouraged about the future

□ I feel discouraged about the future

□ I feel I have nothing to look forward to

□ I feel that the future is hopeless and that things cannot improve

3. Sense of failure

□ I do not feel like a failure

□ I feel I have failed more than the average person

□ As I look back on my life all I can see is a lot of failures

□ I feel I am a complete failure as a person (parent, husband, wife)

1. Lack of satisfaction

□ I am not particularly dissatisfied

□ I don't enjoy things the way I used to

□ I don't get satisfaction out of anything any more

□ I am dissatisfied with everything

1. Guilty feeling

□ I don't feel particularly guilty

□ I feel bad or unworthy a good part of the time

□ I feel bad or unworthy practically all the time now

□ I feel as though I am very bad or worthless

6.Sense of punishment

□ I don't feel I am being punished

□ I have a feeling that something bad may happen to me

□ I feel I am being punished or will be punished

□ I feel I deserve to be punished

7. Self hate

□ I don't feel disappointed in myself

□ I am disappointed in myself

□ I am disgusted with myself

□ I hate myself

8. Self accusations

□ I don't feel I am any worse than anybody else

□ I am very critical of myself for my weaknesses or mistakes

□ I blame myself for everything that goes wrong

□ I feel I have many bad faults

9. Self-punitive wishes

□ I don't have any thoughts of harming myself

□ I have thoughts of harming myself but I would not carry them out

□ I feel I would be better off dead

□ I would kill myself if I could

10. Crying spells

□ I don't cry any more than usual

□ I cry more now than I used to

□ I cry all the time now. I can't stop it

□ I used to be able to cry but now I can't cry at all even though I want to

11. Irritability

□ I am no more irritated now than I ever am

□ I get annoyed or irritated more easily than I used to

□ I feel irritated all the time

□ I don't get irritated at all at the things that used to irritate me

12. Social withdrawal

□ I have not lost interest in other people

□ I am less interested in other people now than I used to be

□ I have lost most of my interest in other people and have little feeling for them

□ I have lost all my interest in other people and don't care about them at all

13. Indecisiveness

□ I make decisions about as well as ever

□ I am less sure of myself now and try to put off making decisions

□ I can't make decisions any more without help

□ I can't make any decisions at all any more

14. Body image

□ I don't feel I look any worse than I used to

□ I am worried that I am looking old or unattractive

□ I feel that there are permanent changes in my appearance and they make me look unattractive

□ I feel that I am ugly or repulsive looking

15. Work inhibition

□ I can work about as well as before

□ I don't work as well as I used to

□ I have to push myself very hard to do anything

□ I can't do any work at all

16. Sleep disturbance

□ I can sleep as well as usual

□ I wake up more tired in the morning than I used to

□ I wake up 1-2 hours earlier than usual and find it hard to get back to sleep

□ I wake up early every day and can't get more than 5 hours sleep

17. Fatigability

□ I don't get any more tired than usual

□ I get tired more easily than I used to

□ I get tired from doing anything

□ I get too tired to do anything

18. Loss of Appetite

□ My appetite is no worse than usual

□ My appetite is not as good as it used to be

□ My appetite is much worse now

□ I have no appetite at all any more

19. Weight Loss

□ I haven't lost much weight, if any, lately

□ I have lost more than 5 pounds

□ I have lost more than 10 pounds

□ I have lost more than 15 pounds

20. Somatic Preoccupation

□ I am no more concerned about my health than usual

□ I am concerned about aches and pains or upset stomach or constipation or other unpleasant feelings in my body

□ I am so concerned with how I feel or what I feel that it's hard to think of much else

□ I am completely absorbed in what I feel

21. Loss of Libido

□ I have not noticed any recent change in my interest in sex

□ I am less interested in sex than I used to be

□ I am much less interested in sex now

□ I have lost interest in sex completely

**Short Screening Scale for** **Post-traumatic stress disorder (PTSD-7)**

1. Did you avoid being reminded of this experience by staying away from certain places, people or activities?

□ Yes

□ No

2. Did you lose interest in activities that were once important or enjoyable?

□ Yes

□ No

3. Did you begin to feel more isolated or distant from other people?

□ Yes

□ No

4. Did you find it hard to have love or affection for other people?

□ Yes

□ No

5. Did you begin to feel that there was no point in planning for the future?

□ Yes

□ No

6. After this experience were you having more trouble than usual falling asleep or staying asleep?

□ Yes

□ No

7. Did you become jumpy or get easily startled by ordinary noises or movements?

□ Yes

□ No

**Young’s Internet Addiction Test**

1. How often do you find that you stay on-line longer than you intended?

□ Rarely

□ Occasionally

□ Frequently

□ Often

□ Always

2. How often do you neglect household chores to spend more time on-line?

□ Rarely

□ Occasionally

□ Frequently

□ Often

□ Always

3. How often do you prefer the excitement of the Internet to intimacy with your partner?

□ Rarely

□ Occasionally

□ Frequently

□ Often

□ Always

4. How often do you form new relationships with fellow on-line users?

□ Rarely

□ Occasionally

□ Frequently

□ Often

□ Always

5. How often do others in your life complain to you about the amount of time you spend on-line?

□ Rarely

□ Occasionally

□ Frequently

□ Often

□ Always

6. How often do your grades or school work suffer because of the amount of time you spend on-line?

□ Rarely

□ Occasionally

□ Frequently

□ Often

□ Always

7. How often do you check your e-mail before something else that you need to do?

□ Rarely

□ Occasionally

□ Frequently

□ Often

□ Always

8. How often does your job performance or productivity suffer because of the Internet?

□ Rarely

□ Occasionally

□ Frequently

□ Often

□ Always

9. How often do you become defensive or secretive when anyone asks you what you do on-line?

□ Rarely

□ Occasionally

□ Frequently

□ Often

□ Always

10. How often do you block out disturbing thoughts about your life with soothing thoughts of the Internet?

□ Rarely

□ Occasionally

□ Frequently

□ Often

□ Always

11. How often do you find yourself anticipating when you will go on-line again?

□ Rarely

□ Occasionally

□ Frequently

□ Often

□ Always

12. How often do you fear that life without the Internet would be boring, empty, and joyless?

□ Rarely

□ Occasionally

□ Frequently

□ Often

□ Always

13. How often do you snap, yell, or act annoyed if someone bothers you while you are on-line?

□ Rarely

□ Occasionally

□ Frequently

□ Often

□ Always

14. How often do you lose sleep due to late-night log-ins?

□ Rarely

□ Occasionally

□ Frequently

□ Often

□ Always

15. How often do you feel preoccupied with the Internet when off-line, or fantasize about being on-line?

□ Rarely

□ Occasionally

□ Frequently

□ Often

□ Always

16. How often do you find yourself saying “just a few more minutes” when on-line?

□ Rarely

□ Occasionally

□ Frequently

□ Often

□ Always

17. How often do you try to cut down the amount of time you spend on-line and fail?

□ Rarely

□ Occasionally

□ Frequently

□ Often

□ Always

18. How often do you try to hide how long you’ve been on-line?

□ Rarely

□ Occasionally

□ Frequently

□ Often

□ Always

19. How often do you choose to spend more time on-line over going out with others?

□ Rarely

□ Occasionally

□ Frequently

□ Often

□ Always

20. How often do you feel depressed, moody, or nervous when you are off-line, which goes away once you are back on-line?

□ Rarely

□ Occasionally

□ Frequently

□ Often

□ Always
